# Comment on ‘Two new compsognathid-like theropods show diversified predation strategies of theropod dinosaurs’ by Qiu *et al*.

**DOI:** 10.1093/nsr/nwaf131

**Published:** 2025-04-02

**Authors:** Christophe Hendrickx

**Affiliations:** Dinosauria Lab, Fundación Miguel Lillo, Argentina; Unidad Ejecutora Lillo, CONICET-Fundación Miguel Lillo, Argentina

Frozen in time by multiple volcanic eruptions, the exquisitely preserved (Lagerstätte) fossils from the Barremian (Lower Cretaceous) Yixian Formation of western Liaoning offer a remarkable glimpse into an incredibly rich ecosystem that thrived in what is now northeastern China ∼125 million years ago [1]. Together with fossils from the Huajiying and Jiufotang formations, they form an assemblage that comprises >60 species of plants, thousands of species of invertebrates and >90 species of vertebrates known as the Jehol Biota. One of the most famous species from the Jehol Biota is *Sinosauropteryx prima*, which provided the first evidence of protofeathers in a non-avian dinosaur (i.e. dinosaurs without their avian descendants, the birds) in 1996 [2]. Named by Qiang Ji and Shu'an Ji [3], *Sinosauropteryx* was initially thought to be the earliest fossil bird and the first representative of the new family “Sinosauropterygidae.” It was, however, reclassified as a member of the Compsognathidae—a small radiation of basally branching coelurosaur theropods encompassing small-bodied (<3 m in length) bipedal dinosaurs that mainly inhabited Eurasia during the Late Jurassic and the Early Cretaceous [4]. The feeding ecology of compsognathids is among the best documented in non-avian theropod dinosaurs, with most species preserving stomach content and, in one exceptional case, ingested skeletal remains within extraordinarily preserved internal organs [5]. Direct evidence of their diet revealed that these small dinosaurs were opportunistic carnivores that fed on a wide array of prey items such as fish, lizards, birds and mammals [6]. The classification of compsognathids, however, is more obscure, with a recent study suggesting that they probably form an artificial grouping of immature individuals that belong to other theropod clades [7]. Compsognathid species indeed appear to be mostly, if not exclusively, represented by skeletally immature specimens that, other than their small size, show several traits that are seen in juvenile theropods such as unserrated teeth [7].

Qiu *et al.*’s [8] recent study on two new compsognathid-like theropods from the Yixian Formation provides support for a radiation of early diverging coelurosaurs that may have been restricted to Northeastern Asia. The authors describe two nearly complete and relatively well-preserved specimens that represent two new coelurosaur species classified either among Compsognathidae or Sinosauropterygidae (the latter being a clade of compsognathid-like coelurosaurs predominantly from the Yixian Formation), depending on the datamatrix that is used in the phylogenetic analysis. Gut contents combined with the morphology of the skull, teeth, neck and manus led Qiu *et al.* [8] to propose that these two new coelurosaurs and a third closely related species had distinct hunting strategies. The first, *Huadanosaurus* (∼1 m), was suggested to be a nocturnal predator that swallowed as a whole small prey items such as mammals. The second, *Sinosauropteryx* (∼1.2 m), likely hunted during the day and dismembered gracile prey such as lizards and insects. The third, *Sinocalliopteryx* (2.4 m), fed on larger animals such as dromaeosaurids and birds. The authors explained these distinct feeding ecologies among closely related and coeval carnivores, and the general diversification of theropods from the Jehol Biota, by the presence of separate ecosystems that were created by the formation of small and isolated rift basins during the destruction of the North China craton.

Interestingly, the two new species described by Qiu *et al.* [8] were identified as juveniles and it is regrettable that the authors did not provide stronger arguments against the possibility that they instead represent immature individuals of already-known species. Indeed, besides the three compsognathids listed above, the Jehol Biota also includes the basally branching tyrannosauroids *Dilong, Sinotyrannus* and *Yutyrannus*—three distant relatives of the eponymous *Tyrannosaurus*—that are phylogenetically close to compsognathids. An alternative hypothesis is that the two newly described species possibly represent juvenile tyrannosauroids such as those of the large-bodied (9–10 m) and most certainly mature *Sinotyrannus* and *Yutyrannus*, whose feeding strategy may have changed during growth. Such an ontogenetic dietary shift was already proposed in two North American tyrannosaurids based on mandibular biomechanical properties and tooth morphology [9]. Ontogenetical changes in non-avian theropods remain vastly poorly understood, leading to heated debates about the validity of some taxa such as *Nanotyrannus*, which is probably a juvenile *Tyrannosaurus* [10]. Only the discovery of additional early diverging coelurosaurs of well-constrained ontogenetical stages from the Jehol Biota, combined with a much better understanding of theropod ontogeny, will enable validation of the separation of several coeval compsognathid-like species from this exceptionally preserved ecosystem and maintain the validity of Compsognathidae and Sinosauropterygidae as non-artificial monophyletic groupings.

***Conflict of interest statement*.** None declared.
